# Identification of New Single Nucleotide Polymorphisms Potentially Related to Small Ruminant Lentivirus Infection Susceptibility in Goats Based on Data Selected from High-Throughput Sequencing

**DOI:** 10.3390/pathogens13100830

**Published:** 2024-09-25

**Authors:** Magdalena Materniak-Kornas, Katarzyna Ropka-Molik, Katarzyna Piórkowska, Joanna Kowalik, Tomasz Szmatoła, Jacek Sikora, Aldona Kawęcka, Jacek Kuźmak

**Affiliations:** 1 Department of Biochemistry, National Veterinary Research Institute, 24-100 Pulawy, Poland; 2 Department of Animal Molecular Biology, National Research Institute of Animal Production, Krakowska 1, 32-083 Balice, Poland; 3 Center for Experimental and Innovative Medicine, University of Agriculture in Krakow, Rędzina 1c, 30-248 Krakow, Poland; 4 Department of Sheep and Goat Breeding, National Research Institute of Animal Production, Krakowska 1, 32-083 Balice, Poland

**Keywords:** small ruminant lentiviruses (SRLVs), goats, susceptibility to infection, resistance to infection, single nucleotide polymorphism

## Abstract

Small ruminant lentivirus (SRLV) infections are spread in the flocks of sheep and goats all over the world, causing economic loss. Although only a fraction of infected animals develop disease symptoms, all of them may shed the virus, causing uncontrolled spread of the infection. Antibodies against the virus can be detected in the blood of infected animals and are the main marker of infection. Additionally, in most infected animals, proviral DNA can also be detected, but at different levels. Due to the lack of treatment or vaccines, the most effective strategy to prevent SRLV infections are control programmes introduced by several countries based on the elimination of seropositive individuals from the flock. An alternative approach, which has currently become the rationale, is an identification of host factors which may predispose certain individuals or breeds to resistance or susceptibility to small ruminant lentivirus infection. In our work, attention was paid to goats of the Carpathian breed infected with SRLV. Available RNA-seq results from the blood of 12 goats with a determined level of SRLV proviral load were used to analyse single nucleotide polymorphisms (SNPs) by the variant calling method. Six SNPs within five genes (*POU2AF1*, *BCAT2*, *TMEM154*, *PARP14*, *UBASH3A*) were selected for genotyping to determine their association with the level of small ruminant lentivirus proviral DNA in a group of 60 goats. Interestingly, in seronegative individuals, only the TT genotype of the *PARP14* gene was observed, while the *TMEM154* CC genotype was found only in seropositive goats. Both genes may be considered potential markers for resistance/susceptibility to SRLV infection. In contrast, polymorphisms identified in *POU2AF1* and *UBASH3A* genes seemed to be deleterious for respective protein functions; therefore, these genes are less likely to be recognised as resistance/susceptibility markers of SRLV infection.

## 1. Introduction

Small ruminant lentiviruses (SRLVs) are a heterogeneous group of viruses within the family *Retroviridae,* comprising several genotypes (A, B, C, E and D, which is still questionable) infecting sheep and goats [1]. Historically, genotype A includes maedi-visna virus (MVV)-like strains, and genotype B includes caprine arthritis-encephalitis virus (CAEV)-like strains. According to many reports from the past two decades, it is known that SRLVs infect the monocytes and macrophages of sheep and goats, causing cross- and superinfections, which lead to persistent infection and chronic debilitating disease [2]. In about 30% of infected goats, SRLV causes emaciation, progressive arthritis, and mastitis, while in a similar percentage of sheep, it manifests itself with a progressively wasting disease called maedi-visna. The major routes of SRLV transmission are the consumption of colostrum and milk from infected dams and long-term direct contact between individuals [3,4], while intrauterine transmission is regarded as a possible but sporadic way of infection [5]. SRLV infections are spread worldwide, and their economic impact on the production and welfare of goats and sheep is significant [4]. Since no effective vaccine nor treatment against SRLV infection is available, the only way to prevent the spread of the virus seems to be through control programmes. The most effective practice is culling infected animals based on positive serology. An alternative approach implemented in herds with high seroprevalence is the selection of progeny from seronegative dams and the artificial feeding of negative offspring. Unfortunately, such practices have some disadvantages, such as the variation in serological response in different animals in flocks and high genetic variability, which hampers serodiagnostics [6,7]. Furthermore, they are expensive and time-consuming; therefore, new, more effective control measures are required. During the last few years, many studies have focused on the host factors that determine the resistance of individual animals or breeds to SRLV infection. Finding such markers may lead to identifying and selecting individuals naturally resistant to SRLV. Such a phenomenon is known for different pathogens, but the mechanisms typical of SRLV in sheep and goats are still under investigation.

Since SRLV infects sheep and goats, showing sustained antibody response and suggesting virus replication at a certain level, it is evident that typical species-specific barriers, based on some restriction factors, do not work properly in this case. However, even in flocks with a high SRLV prevalence, there are individuals with low proviral loads and even negative ones. Therefore, a low SRLV proviral load has been proposed as a biomarker of natural resistance to infection [7]. The increased expression of genes encoding restriction factors responsible for facing lentiviral infections like *TRIM5α* (tripartite motif protein 5 alpha), *APOBEC3* (apolipoprotein B editing complex 3), and *Tetherin* have been described as the main factors that can block the development of infection and the transmission of the virus through their negative impact on its replication [7]. The second approach focuses on the role of specific single nucleotide polymorphisms (SNPs) in various genes like *TMEM154* (transmembrane protein 154), *MYO88* (MYD88 Innate Immune Signal Transduction Adaptor), *TLR*s (Toll-like receptors), *CCR5* (chemokine (C–C motif) Receptor 5), and *TRIM5α* [7,8,9,10] as factors which affect the functions of the proteins they encode; these are responsible for virus–cell interactions, significantly reducing the level of proviral DNA, presumably protecting low proviral carriers against virus shedding [7]. Interestingly, most studies focus on sheep, while so far, only a few reports have described identifying genes suspected to be markers of SRLV resistance in goats [7,11,12,13]. Unfortunately, polymorphisms suggested to predispose sheep to SRLV infection resistance were not confirmed in goats. White and co-workers showed that the *CCR5* gene with a deletion of four nucleotides in the promoter region correlated with an almost four-fold reduction in SRLV proviral DNA levels in sheep, making these animals less susceptible to SRLV infection. However, data obtained in the goat study differed. The mutation of g.1059T, found in the *CCR5* gene in goats, correlated with an almost three-fold increase in the provirus copy number, determined that these individuals were more susceptible to SRLV infection [13]. In the previous study, 9 single nucleotide polymorphisms (SNPs), identified in the coding sequences of *TLR* 7 and 8 genes, showed a correlation with small ruminant lentivirus proviral load [11].

In the present study, we aim to extend the analysis of SNPs in goat genes based on available RNA-seq results in the context of their association with SRLV infection. Correlating the results of SNP analysis with the level of SRLV proviral DNA can help select genes with a potential link to the susceptibility of goats to infection with these viruses.

## 2. Materials and Methods

### 2.1. Animals and Blood Samples

Jugular vein blood was collected from 60 goats into tubes with EDTA (for DNA isolation) and without EDTA (for serological testing). All goats were from the Carpathian breed and came from the National Research Institute of Animal Production Experimental Station in Odrzechowa. The goats were females at a similar age; all were healthy and kept in one herd in the same environment.

### 2.2. Serological Testing of Serum Samples

The presence of antibodies against SRLV was determined by an enzyme-linked immunosorbent assay (ELISA) (ID Screen MVV/CAEV Indirect Screening test, IDVet, Grabels, France) in the blood serum of 60 goats according to the manufacturer’s recommendations.

### 2.3. Quantification of SRLV Proviral DNA

Genomic DNA was isolated from the peripheral blood leukocytes of 60 goats using the NucleoSpin Blood Quick Pure kit (Machery-Nagel, Düren, Germany). The proviral DNA of SRLV was quantified by a qPCR assay using Rotor-Gene Q (Qiagen, Hilden, Germany), with primers and a probe specific for the SRLV A5 subtype, the presence of which in this herd had been previously confirmed [14]. The following primers were used in the qPCR reaction: CA5F (5′-TGGGAGTAGGACAAACAAATCA-3′), CA5R (5′-TGACATATGCCTTTACTGCTCTC-3′) and the CA5P probe (5′-6-FAM-TCACCCATTGTAGCATAGCTGCC-BHQ-1-3′) [12]. The standard curve was determined using 10-fold serial dilutions (from 10^6^ to 100 copies) of plasmid DNA containing a 625 bp *gag* gene fragment of SRLV A5 as the template. Amplification was carried out at a total volume of 20 µL under the following conditions: pre-incubation and polymerase activation at 95 °C for 15 min, 45 cycles of 94 °C for 60 s, and 60 °C for 60 s. The reaction mixture contained 10 µL of a 2× QuantiTect Probe PCR buffer (Qiagen, Hilden, Germany), 400 nM of each primer, 200 nM of a specific probe, and 5 µL of the DNA template. All samples were tested in duplicate, and the results were quantified as the average number of copies per 500 ng of genomic DNA for each goat.

### 2.4. SNPs Identification Based on RNA-Seq Data

The SNP detection was performed based on 12 whole blood transcriptomes of Carpathian goats. The high-throughput data from previous research GSE168160 focused on gene expression modifications under different proviral loads of small ruminant lentiviruses in goats grouped as having a high and low proviral load (HPL and LPL) [12].

The raw reads were checked for quality assessment with the use of FastQC software (v. 0.12) [15], followed by a trimming procedure which led to removing reads that did not maintain the following criteria: <20 phred quality, reads shorter than 35 bp, and the presence of adapters (Flexbar) [16]. After the trimming procedure, the filtered reads were mapped to the goat *Capra hircus* ARS1 reference genome with the use of STAR software (v. 2.7.11) [17]. In the first step of the variant calling approach, Picard tools [18] were used to mark duplicated reads, possibly resulting from a PCR bias. Then, we used Freebayes software (v. 1.3.8) to call SNP and Indel variants, with an option not to call the complex events selected. The filtration step, maintained with the use of the GATK Variant Filtration tool [19], was used to remove variants that did not pass the following criteria: variant quality > 30 (QT > 30); coverage > 10 (DP > 10); Fisher strand < 60 (FS < 60); Strand Odds Ratio < 3 (SOR < 3); and RMS Mapping Quality > 40 (MQ > 40).

Next, SNPs with the different allele frequencies between goat groups with a high (HPL) and low (LPL) proviral load were selected. The selection criteria to consider specific variants was the allele distribution between the analysed groups, with at least 67% of allele frequency in the first group (four alleles per six individuals in one group), with a similar frequency in the second group, but with an alternate allele.

### 2.5. Polymorphism Selection and Genotyping

Based on information on RNA-seq SNPs, 6 mutations within 5 genes (*POU2AF1, BCAT2*, *TMEM154*, *PARP14*, *UBASH3A*) were selected. For genotyping, primers were designed in the Primer3 (v. 0.4.0) tool based on the reference sequence shown in Table 1. Mutations in *POU2AF1*, *BCAT2*, and *TMEM154* genes were detected by the PCR-RFLP method; the NEBcutter V2.0 tool was used for setting restriction enzymes (Table 2). Variants for *PARP14* and *UBASH3A* genes were genotyped using Sanger sequencing. Genotyping included all 60 goats.

For sequencing, the PCR amplification products were obtained using the AmpliTaq Gold™ 360 Master Mix (Thermo Fisher Scientific, Waltham, MA, USA) and purified using the enzyme mixture EPPiC (A&A Biotechnology, Gdynia, Poland). According to the manufacturer’s protocol, Sanger sequencing was performed using the BigDye™ Terminator v3.1 Cycle Sequencing Kit (Thermo Fisher Scientific, Waltham, MA, USA) and BigDye XTerminator™ Purification Kit (Thermo Fisher Scientific). The amplicons were separated on a 3500xL Genetic Analyzer (Applied Biosystems, Thermo Fisher Scientific). Data were analysed using Data Collection Software (Applied Biosystems). All identified variants are previously known and had an rs number (available in Ensembl).

### 2.6. Statistical Analysis

A simple linear model approach was performed using a *t*-test to identify the association between genotypes and proviral loads. In this analysis, SNP was used as a classification variable, and proviral load values were used as analysis variables. Quantitative results given as the means ± SD presented specific significant differences between animals within given genotype groups.

The genotype distribution of the SNPs was tested for deviations from the Hardy–Weinberg equilibrium (HWE) using the Court lab–HW calculator, including χ^2^ analysis (*p*-value < 0.05).

## 3. Results

### 3.1. Serological Status of Goats

Among the serum samples collected from 60 goats, 56 showed the presence of specific antibodies against SRLV, while the remaining 4 were seronegative.

### 3.2. Whole-Transcriptome SNP Identification Results

**Figure 1:**
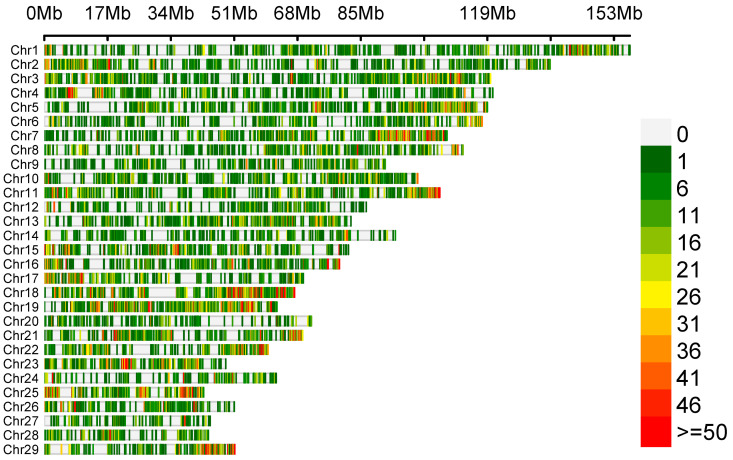
Density plot depicting the distribution of identified genetic variants.

The variant calling allowed us to detect 65,535 polymorphisms, of which only 57 SNPs met the selection criteria of significant differences in allele frequency between the goat groups with high (HPL) and low (LPL) proviral loads. The density of identified genetic variants is illustrated in Figure 1. The number of variants is displayed in 0.1 Mb intervals across the entire genome.

Out of 57 SNPs, 22 were localised on the 18th chromosome (between 26,127,829 and 6,127,829 bp), and the others were localised on chromosomes 1 (n = 13), 11 (n = 6), 12 (n = 1), 13 (n = 5), 15 (n = 3), and 17 (n = 7) in genes and in intergenic regions (Table S1).

**Figure 2:**
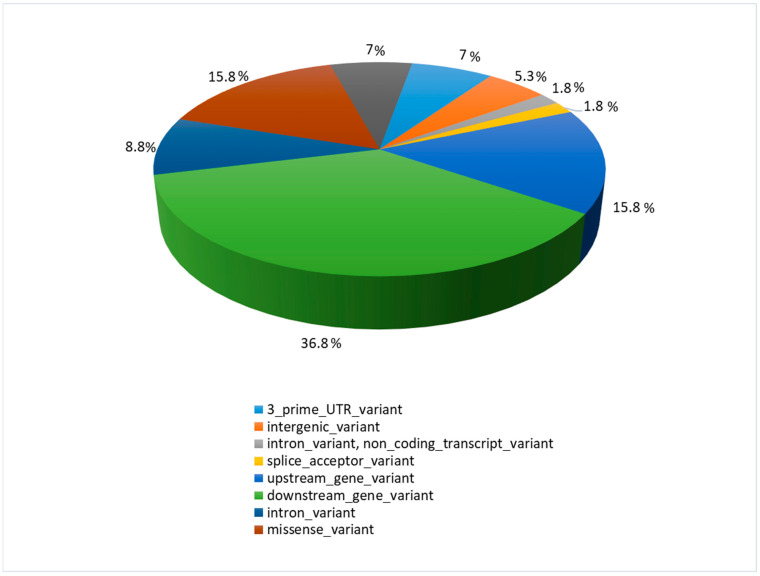
The function and location of identified polymorphisms with their percentage share.

Most detected polymorphisms were downstream gene variants (ds) (36.8%), while upstream gene variants (up) constituted 15.8%, similar to the highest effect—the missense variants (ms) (15.8%) (Figure 2).

As shown in Gene Ontology analysis (David software v. 6.8), 8 out of 29 genes with significant gene variants belonged to Immune Systems Process GO:0002376 (*BCL3*; *PKNOX1*; *POU2AF1*; *RASSF2*; *IL18R1*; *MCM3AP*; *PARP14*; *UBASH3A*).

### 3.3. Detected SNPs, Allele and Genotype Frequency

For genotyping, 6 SNPs within five genes (*POU2AF1*, *BCAT2*, *TMEM154*, *PARP14, UBASH3A*) were selected based on the gene’s function (their involvement in the immune response of the host) and polymorphism types (SNPs with the potential for the highest effect on protein functions) (Table 2). These underwent association analysis with the proviral load. Restriction enzymes designed for genotyping are shown in Table 2.

Regarding the *PARP14* gene locus, seronegative individuals were observed only for TT genotypes, and the *TMEM154* CC group was free of seronegative goats. On the other hand, the GERP test indicated that the *POU2AF1* ds mutation, *UBASH3A* missense variant, and silent variant should be the most deleterious for protein function; therefore, they should be considered with more attention. The analysis of frequency for selected polymorphisms showed that in Carpathian goats in loci, *BCAT2*_T>C and *TMEM154*_T>C alternate alleles prevail. Moreover, all investigated loci were consistent with the HW equilibrium, but in some cases, in particular the groups with a low number of individuals (<5), the HWE test was inaccurate (Table 3).

### 3.4. Association between SNPs and Provirus Copy Number

The level of SRLV proviral DNA was determined in blood leukocytes obtained from 56 serologically positive goats and four negative individuals by qPCR assay. In 4 of the 56 positive goats, the level of proviral DNA could not be determined, while in the remaining 52 goats, the provirus copy number ranged between 0.2 and 7420 copies per 500 ng of genomic DNA. The proviral load was checked for some individuals twice in the experiment, including those with outlier results. The copy number of SRLV in individual animals differed a little in time, but it was always in the same range.

Initially, when RNA-seq data were analysed, only three goats were uninfected with SRLVs [12]; therefore, it was impossible to create a control group with an equivalent number of animals to those that were serologically positive and compare allele and genotype frequencies between infected and uninfected goats. Therefore, in this study, only associations between identified SNPs and the provirus copy number of SRLVs were estimated for all investigated genes.

**Figure 3:**
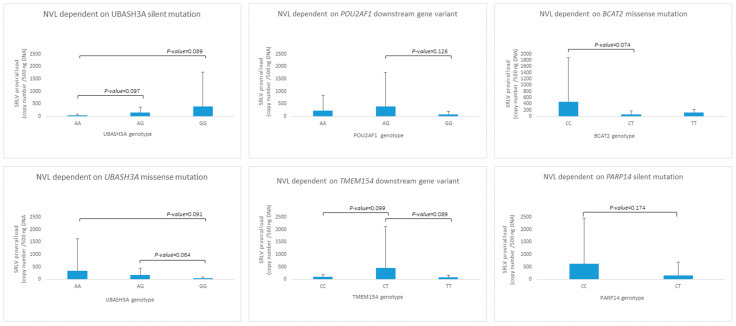
The genotype distribution for SNPs detected in UBASH3A, POU2AF1, BCAT2, TMEM154 and PARP14 genes.

Although association analysis did not show any statistically significant relationship between the provirus copy number and investigated SNPs, trends were observed in some cases (Figure 3). The silent variant for genotype AA in *UBASH3A* was observed only in two individuals, but the relationship between AA < AG < GG and proviral load (*p* < 0.1) was noted. A similar observation was made for the *UBASH3A* missense mutation, but in the opposite direction, AA > AG > GG. *POU2AF1, BCAT2,* and *TMEM15* loci heterozygotes seem to be distinct from both homozygotes in the context of proviral load; nevertheless, only trends were observed (*BCAT2* and *TMEM15*, *p*-value < 0.1). In the *PARP14* locus, individuals with the CC genotype were not observed; however, the GERP test did not indicate harmfulness for its high protein function.

## 4. Discussion

In the present study, we attempted to identify markers predicting the resistance or susceptibility of goats to SRLV infection based on the search for significant gene variants within RNA–seq data. For testing, polymorphisms found in genes engaged in immune response and associated with proviral load were selected, including numerous infected animals from the same flock. The presented method of using high-throughput sequencing data showed the optimal approach for identifying new polymorphisms at the whole transcriptome level. Moreover, detecting differences in allele frequency between goat groups with a high (HPL) and low (LPL) proviral load allowed us to pinpoint the genes potentially related to susceptibility/resistance in SRLV infection.

One of the interesting polymorphisms was identified in the downstream region of the *TMEM154* gene (T>C, 17: 65805153). The *TMEM154* gene, which encodes transmembrane protein 54, has already been reported in the context of numerous polymorphisms, some of which appear to be significantly related to the susceptibility of sheep to SRLV infection [9,10]. Previous studies have shown that sheep carrying haplotype 3, encoding glutamic acid (E) at position 35 and asparagine (N) at position 70 of the TMEM154 protein, and haplotype 2, encoding isoleucine (I) at position 70 of this protein, are highly susceptible to SRLV infection. In contrast, individuals carrying haplotype 1, encoding lysine (K) at position 35 of the protein, are considered much less vulnerable to infection. Moreover, it is interesting to note that in our study, the TT genotype in the *TMEM154* gene was observed only in SRLV seropositive animals.

Another polymorphism was identified in the *POU2AF1* gene (also called *OCA-B*, *OBF-1*, and *BOB.*1), which has been described mainly as lymphocyte-specific, where it functions as a co-activator of the octameric transcription factors OCT1 and OCT2 in regulating the expression of immunoglobulin and host defence-related genes [20,21]. POU2AF1 has no intrinsic DNA-binding activity but recognises the POU domain of the factors OCT1 and OCT2 and plays an essential role in B lymphocyte responses to antigens [22,23]. However, reports have suggested that POU2AF1 may have a defensive function in cells other than lymphocytes, including the respiratory epithelium [24]. In vitro studies have shown that the infection of such cells with recombinant lentivirus causes the upregulation of *POU2AF1* gene expression, which, in turn, induces the upregulation of the expression of genes involved in the immune defence response to infections, such as MX1, IFIT3, IFITM and HLA-DRA, ID2, ID3, IL6, BCL6, which are directly under the control of POU2AF1. Given the potential function of the *POU2AF1* gene as a regulator of immune response, the detection of its downstream mutation in some goats, likely with a deleterious impact on protein function may be relevant to the susceptibility/resistance of individuals to SRLV infection and is worth further analysis.

The *PARP14* gene encodes poly ADP-ribose polymerase 14, which belongs to the family of poly(ADP-ribose) polymerase (PARP) proteins and is involved in many processes, from cell differentiation and DNA repair to transcriptional control and pro-inflammatory signalling pathways [25]. PARP14 selectively binds to STAT6 and regulates the expression of interleukin 4 (IL-4)-related genes [26]. Experiments on murine and human cells also indicate the role of PARP14 in the induction of type I interferon production in response to bacterial and viral infections [27,28]. Several reports have shown that the induction of PARP proteins, including PARP14, can result in the inhibition of virus replication [28,29,30,31]. The role of PARP14 in antiviral defence seems to also be supported by phylogenetic analysis, which showed that *PARP* mutation rates have positive selection in the catalytic domains of PARPs, which may suggest a connection between antiviral response and the activity of multiple PARPs [32,33]. In light of these findings, our observation that only one genotype (TT) of the *PARP14* locus was noted in seronegative goats makes this protein a very interesting candidate as a potential marker of goats’ susceptibility to SRLV infection.

Another gene in which two polymorphisms have been identified is *UBASH3A* (the ubiquitin-associated and SH3 domain containing A) [34]. This protein belongs to the ubiquitin ligand family of T cells and may negatively regulate T cell signal transduction [35]. Previous studies have indicated whether genetic variants in the *UBASH3A* gene have been associated with human susceptibility to autoimmune diseases, such as diabetes, rheumatoid arthritis, and systemic lupus erythematosus [36,37]. Thus, the polymorphisms identified in infected goats may have some connection to the course of SRLV infection.

The missense polymorphism was also identified in the *BCAT2* gene encoding an enzyme, branched-chain amino acid aminotransferase, which is involved in the biosynthesis of all branched-chain amino acids (leucine, isoleucine, and valine) and was identified to be down-regulated in patients suffering from tuberculosis (TB) [38,39]. Furthermore, BCAT2 has been described mainly as an important progressive factor of different kinds of cancer, including pancreatic and lung cancer, bladder cancer, as well as other diseases like Alzheimer’s disease or myeloid leukaemia [40,41] due to its mediating role in the regulation of metabolic-related pathways [42]. However, recent studies on bladder cancer have shown the immunosuppressive role of BCAT2 through its inhibitory impact on cytotoxic lymphocyte recruitment by restraining activities of proinflammatory cytokine/chemokine-related pathways and the T-cell chemotaxis pathway [41]. In this context, the *BCAT2* expression level was even tested in forecasting a curative effect of bladder cancer-guided immunotherapy [41]. Considering the undisputed but not yet fully understood role of BCAT2 in immunological processes in cancer and TB infection, one may not exclude its involvement during SRLV infection and its possible role as a susceptibility marker.

## 5. Conclusions

The identification of relevant genes and whether their polymorphisms are associated with goat susceptibility to infection is an important step toward reducing SRLV transmission in goats. In the presented study, only trends suggesting the relationship between gene variants and the level of SRLV proviral DNA were identified. One of these genes was described previously to be associated with the level of proviral DNA in SRLV-infected sheep; thus, it seems to be involved in the host response to infection. The obtained results might also indicate other specific genes that, due to their engagement in immunological processes, may determine the susceptibility/resistance of goats to SRLV infection. However, further analysis is required for the validation of our results on a larger group of goats, especially the inclusion of a group of uninfected animals, to compare allele and genotype frequencies between infected and uninfected goats. In addition, the use of animals representing breeds other than the Carpathian should also be taken into account in such studies.

## Figures and Tables

**Table 1:** Primers used for amplification of selected gene polymorphisms.

Reference Sequence	Sequences of Primers (5′–3′)	Orientation	Size	Identified SNP Based on RNA-Seq Data (HGVS Name)
POU2AF1 ENSCHIG00000020104	TGGTATCTGCCTCAAGTCCC	F	149 bp	rs666106235 (NC_030822.1:g.60734296G>A)
	GACTCCCTTCTCTCTTGCCT	R		
BCAT2 ENSCHIG00000008019	GTACAGGATTTGGTGCACGG	F	155 bp	rs644972268 (NC_030825.1:g.56430949T>C)
	AGGTTATTTGTCTCGCCCCA	R		
TMEM154 ENSCHIG00000026757	ATTTCTCTGTCACCTGGCCA	F	155 bp	rs654984114 (NC_030824.1:g.65805153T>C)
	AGACAGCAAACAAAGCAAGTATT	R		
UBASH3A ENSCHIG00000022525	GAGGAAGGAAAATGGGAGTTGG	F	150 bp	rs668378283 (NC_030808.1:g.142319001A>G)
	TCTGCGGAGTCCCTTCTC	R		rs658091735 (NC_030808.1:g.142318972G>A)
PARP14 ENSCHIG00000018391	CGGGTACTCACTGGATGCTA	F	203 bp	rs664150071 (NC_030808.1:g.66713881C>T)
	TCTGCAAAGGTTACCAAAATGTT	R		

F—forward; R—reverse.

**Table 2:** Genotyping methods for 6 SNPs within 5 goat genes.

Gene Name	loc	Position	Genotyping Method	Variant Type	Restriction Enzyme	rs	HGVS Nomenclature	GERP
*POU2AF1*	15	60,734,296	PCR-RFLP	ds	DraI	rs666106235	NC_030822.1:g.60734296G>A	−6.03
*BCAT2*	18	56,430,949		ms	MnlI	rs644972268	NC_030825.1:g.56430949T>C	0.14
*TMEM154*	17	65,805,153		ds	HgaI	rs654984114	NC_030824.1:g.65805153T>C	−0.84
*UBASH3A*	1	142,319,001	Sanger sequencing	ms	_	rs668378283	NC_030808.1:g.142319001A>G	−6.05
		142,318,972		sv		rs658091735	NC_030808.1:g.142318972G>A	−7.13
*PARP14*	1	66,713,881		sv		rs664150071	NC_030808.1:g.66713881C>T	−1.04

The GERP score is defined as a reduction in the number of substitutions in the multi-species sequence alignment compared to the neutral expectation. ds—downstream gene variant, ms—missense variant, sv—silent variant, and loc—localisation.

**Table 3:** The frequencies of 6 alleles and genotypes in 5 genes.

Gene/Polymorphism	Genotype			Allele		HWE * (*p*-Value)
	ref hom	het	alt hom	ref	alt	
*POU2AF1_G>A*	0.22 (13)	0.5 (30)	0.28 (17)	0.47	0.53	0.97
*BCAT2_T>C*	0.07 (4)	0.40 (24)	0.53 (32)	0.27	0.73	0.86
*TMEM154_T>C*	0.15 (9)	0.55 (33)	0.30 (18)	0.22	0.78	0.33
*UBASH3A_A>G*	0.68 (41)	0.28 (17)	0.04 (2)	0.70	0.30	0.88
*UBASH3A_G>A*	0.56 (34)	0.40 (24)	0.04 (2)	0.60	0.40	0.36
*PARP14_C>T*	0 (0)	0.28 (19)	0.68 (41)	0.23	0.77	0.15

* HWE—Hardy–Weinberg equilibrium. If the *p*-value is < 0.05, it is not consistent with HWE. It is not accurate if there are <5 individuals in any genotype group. In brackets, the number of individuals in the genotype group is given. In the brackets, the number of particular individuals is shown. het—heterozygote, hom—homozygote, alt—alternate, and ref—reference.

## Data Availability

RNA-seq data are available under GEO GSE168160 accession number. Other data generated during and/or analysed during the current study are available on reasonable request.

## Supplementary Materials

The following supporting information can be downloaded at https://www.mdpi.com/article/10.3390/pathogens13100830/s1. Table S1. SNPs identified by variant calling that meet the criteria for significant differences in allele frequency between groups of goats with high and low SRLV proviral load.
