# Supplementary material for: Identification of New Single Nucleotide Polymorphisms Potentially Related to Small Ruminant Lentivirus Infection Susceptibility in Goats Based on Data Selected from High-Throughput Sequencing
Source: Pathogens. 2024 Sep 25;13(10):830. doi: 10.3390/pathogens13100830 (PMC11510762; doi:10.3390/pathogens13100830)
Supplement: Supplementary file 1 [file pathogens-13-00830-s001.zip › pathogens-3126482-supplementary.pdf]

Table S1. SNPs identified by variant calling that meet the criteria for significant differences in allele frequency between groups of goats with high and low SRLV proviral load.

Lp.	Gene accession number	Gene symbol	#CHROM	POS	Consequence	IMPACT
1	ENSCHIG00000008074	*YBEY*	1	145945232	downstream_gene_variant	MODIFIER
2	ENSCHIG00000022525	*UBASH3A*	1	142319001	missense_variant	MODERATE
3	ENSCHIG00000019819	*PKNOX1*	1	142788769	downstream_gene_variant	MODIFIER
4	ENSCHIG00000008616	*PDXK*	1	143388369	synonymous_variant	LOW
5	ENSCHIG00000018391	*PARP14*	1	66713881	missense_variant	MODERATE
6	ENSCHIG00000022369	*PARP14*	1	66713984	downstream_gene_variant	MODIFIER
7	ENSCHIG00000018391	*PARP14*	1	66717952	downstream_gene_variant	MODIFIER
8	ENSCHIG00000025098	*MCM3AP*	1	145896431	missense_variant	MODERATE
9	ENSCHIG00000008859	*GET1*	1	139060878	upstream_gene_variant	MODIFIER
10	ENSCHIG00000026362	-	1	143732353	upstream_gene_variant	MODIFIER
11	ENSCHIG00000012627	-	1	143757516	missense_variant	MODERATE
12	ENSCHIG00000013703	-	1	143826612	downstream_gene_variant	MODIFIER
13	ENSCHIG00000002568	-	1	144318770	upstream_gene_variant	MODIFIER
14	ENSCHIG00000009768	*NDOR1*	11	106039275	downstream_gene_variant	MODIFIER
15	ENSCHIG00000009915	*IL18R1*	11	7067386	downstream_gene_variant	MODIFIER
16	ENSCHIG00000020011	*DDX31*	11	102111615	missense_variant	MODERATE
17	ENSCHIG00000024407	-	11	48786890	3_prime_UTR_variant	MODIFIER
18	-	-	11	99394877	intergenic_variant	MODIFIER
19	ENSCHIG00000018540	-	11	104543634	upstream_gene_variant	MODIFIER
20	ENSCHIG00000021752	-	12	67500851	downstream_gene_variant	MODIFIER
21	ENSCHIG00000001681	*U2*	13	32712990	upstream_gene_variant	MODIFIER
22	ENSCHIG00000024230	*RASSF2*	13	46540683	downstream_gene_variant	MODIFIER
23	ENSCHIG00000013273	*ENTPD6*	13	42100556	downstream_gene_variant	MODIFIER
24	ENSCHIG00000021551	-	13	43310363	downstream_gene_variant	MODIFIER

25	-	-	13	46925978	intergenic_variant	MODIFIER
26	ENSCHIG00000020104	*POU2AF1*	15	60734296	downstream_gene_variant	MODIFIER
27	ENSCHIG00000020550	*COMMD9*	15	16186082	3_prime_UTR_variant	MODIFIER
28	ENSCHIG00000001685	-	15	36042280	missense_variant	MODERATE
29	ENSCHIG00000017615	*VPS37B*	17	18554163	downstream_gene_variant	MODIFIER
30	ENSCHIG00000026757	*TMEM154*	17	65805153	downstream_gene_variant	MODIFIER
31	ENSCHIG00000021555	*GRK3*	17	5688762	intron_variant	MODIFIER
32	ENSCHIG00000014840	*DERL3*	17	103126	downstream_gene_variant	MODIFIER
33	ENSCHIG00000014840	*DERL3*	17	105033	downstream_gene_variant	MODIFIER
34	ENSCHIG00000011258	-	17	28416	missense_variant	MODERATE
35	ENSCHIG00000016381	-	17	858966	downstream_gene_variant	MODIFIER
36	ENSCHIG00000015006	*ZNF45*	18	53172888	3_prime_UTR_variant	MODIFIER
37	ENSCHIG00000020692	*XRCC1*	18	52761033	synonymous_variant	LOW
38	ENSCHIG00000015661	*TMEM150B*	18	63922021	synonymous_variant	LOW
39	ENSCHIG00000003131	*TIMM50*	18	50032718	upstream_gene_variant	MODIFIER
40	ENSCHIG00000000763	*RINL*	18	49484314	upstream_gene_variant	MODIFIER
41	ENSCHIG00000022038	*POLR1G*	18	54080045	downstream_gene_variant	MODIFIER
42	ENSCHIG00000008085	*MED25*	18	57197769	intron_variant	MODIFIER
43	ENSCHIG00000014862	*CLIP3*	18	47527632	downstream_gene_variant	MODIFIER
44	ENSCHIG00000016262	*BCL3*	18	53579618	splice_acceptor_variant	HIGH
45	ENSCHIG00000008019	*BCAT2*	18	56430949	missense_variant	MODERATE
46	ENSCHIG00000025848	-	18	26127829	intron_variant	MODIFIER
47	ENSCHIG00000010568	-	18	37760815	upstream_gene_variant	MODIFIER
48	ENSCHIG00000004478	-	18	51633555	intron_variant,non_coding_transcript_variant	MODIFIER
49	ENSCHIG00000017650	-	18	57586467	upstream_gene_variant	MODIFIER
50	ENSCHIG00000024565	-	18	60966409	downstream_gene_variant	MODIFIER
51	ENSCHIG00000024565	-	18	60968627	missense_variant	MODERATE
52	ENSCHIG00000000547	-	18	62613155	downstream_gene_variant	MODIFIER

53	ENSCHIG00000020885	-	18	64205274	intron_variant	MODIFIER
54	ENSCHIG00000020885	-	18	64213075	intron_variant	MODIFIER
55	ENSCHIG00000005560	-	18	64552477	synonymous_variant	LOW
56	ENSCHIG00000015449	-	18	64570706	3_prime_UTR_variant	MODIFIER
57	-	-	18	64675807	intergenic_variant	MODIFIER
